# Associations of Polymorphisms in Histidine Decarboxylase, Histamine N-Methyltransferase and Histamine Receptor H_3_ Genes with Breast Cancer

**DOI:** 10.1371/journal.pone.0097728

**Published:** 2014-05-16

**Authors:** Gong-Hao He, Jia-Ji Lin, Wen-Ke Cai, Wen-Mang Xu, Zheng-Ping Yu, Sun-Jun Yin, Can-Hu Zhao, Gui-Li Xu

**Affiliations:** 1 Department of Pharmacy, Kunming General Hospital of Chengdu Military Region, Kunming, Yunnan, China; 2 Department of neurology, Tangdu Hospital, Fourth Military Medical University, Xi’an, China; 3 Department of Cardio-Thoracic Surgery, Kunming General Hospital of Chengdu Military Region, Kunming, Yunnan, China; 4 Department of Pathology, Kunming General Hospital of Chengdu Military Region, Kunming, Yunnan, China; 5 Department of Labour Hygiene, College of Military Preventive Medicine, Third Military Medical University, Chongqing, China; University of North Carolina School of Medicine, United States of America

## Abstract

We previously found that genetic polymorphisms in gene coding for histamine H_4_ receptors were related to the risk and malignant degree of breast cancer. The roles of polymorphisms in other histamine-related genes, such as *histidine decarboxylase* (*HDC*), *histamine N-methyltransferase* (*HNMT*) and *histamine H_3_ receptor* (*HRH3*), remain unexplored. The aim of this study is to analyze the clinical associations of polymorphisms in *HDC*, *HNMT* and *HRH3* with breast cancer. Two hundred and one unrelated Chinese Han breast cancer patients and 205 ethnicity-matched health controls were recruited for case-control investigation. Genomic DNA from the participants was extracted and 5 single nucleotide polymorphisms (SNPs) in *HDC*, *HNMT* and *HRH3* were genotyped. We found that polymorphisms of *HNMT* and *HRH3* were irrelevant with breast cancer in the present study. However, the T allele of rs7164386 in *HDC* significantly decreased the risk of breast cancer (adjusted odds ratios [ORs], 0.387; 95% confidence intervals [CIs], 0.208–0.720; *P* = 0.003). Furthermore, for *HDC* haplotypes, the CG haplotype of rs7164386-rs7182203 was more frequent among breast cancer patients (adjusted OR, 1.828; 95% CI, 1.218–2.744; *P* = 0.004) while the TG haplotype was more frequent among health controls (adjusted OR, 0.351; 95% CI, 0.182–0.678; *P* = 0.002). These findings indicated that polymorphisms of *HDC* gene were significantly associated with breast cancer in Chinese Han population and may be novel diagnostic or therapeutic targets for breast cancer. Further studies with larger participants worldwide are still needed for conclusion validation.

## Introduction

It is now gradually recognized that histamine and its related proteins (synthesis and catabolic enzymes or different types of receptors) play important roles in many aspects of breast cancer. For instance, the histamine concentration in neoplasmatic tissues of breast cancer patients was found to be higher than that in unchanged tissues of healthy controls and the activity of histidine decarboxylase (HDC), the only enzyme that catalyzes the formation of histamine, was believed to be related to this phenomenon [1]. Furthermore, knocking out *HDC* was suggested to result in decreasing breast tumor growth in mice [2]. Additionally, one of the histamine catabolic enzymes, histamine N-methyltransferase (HNMT), and histamine H_3_ (HRH3) and H_4_ (HRH4) receptors were also closely involved in proliferation and differentiation of breast cancer according to recent investigations [3]–[5]. Therefore, these histamine-related proteins may be important diagnostic or therapeutic targets of breast cancer and clarifying their relationships with breast cancer risk and development will be one of the important topics in this field.

Meanwhile, certain single nucleotide polymorphisms (SNPs) in genes that encode the abovementioned histamine-related proteins (i.e., *HDC*, *HNMT*, *HRH3* and *HRH4*) were reported to affect the gene expression and function and were associated with risks of diseases [6]–[10]. Therefore, with respect to the notion that genetic factors contribute greatly to the risk and development of breast cancer [11]–[12], polymorphisms of *HDC*, *HNMT*, *HRH3* and *HRH4* are likely to be also associated with breast cancer risk. In our previous investigation, we have found that variants of *HRH4* gene were significantly associated with the risk and malignant degree of breast cancer in Chinese Han populations [10]. However, the associations between polymorphisms of *HDC*, *HNMT*, *HRH3* and susceptibility to breast cancer are still unknown so far and need to be elucidated.

Based on this background, the goal of the present study was to further demonstrate the relationships between certain tag-SNPs or previously reported positive SNPs of *HDC*, *HNMT* and *HRH3* and breast cancer risk using case-control method among Chinese Han population in order to provide better insights into the risk and development of breast cancer.

## Materials and Methods

### Subjects

Two hundred and one randomly selected patients with breast cancer who were admitted to Kunming General Hospital of Chengdu Military Region between 2009 and 2013, and 205 unrelated healthy individuals who had no known medical illness or hereditary disorders and who were not taking any medications were included. Patients who had comorbidity, such as diabetes mellitus, hypertension or any endocrine disorders were excluded from the present study. All of the participants were Han Chinese women living in Kunming city and nearby. Principal clinical characteristics, such as age at diagnosis, body mass index, and menopausal state, were obtained from the interviewer-administered health risk questionnaires. Menopausal status was defined as the date of last menses followed by 12 months of no menses. The clinicopathological variables and prognostic factors, i.e., tumor size, histology, clinical stages, lymph node involvement, hormone receptor (including estrogen receptor and progesteron receptor) status, HER2 status, and p53 status, were obtained from the patients’ medical records. The histologic determinations, including tumor type and disease stage, were performed according to the World Health Organization criteria and the TNM classification system, respectively.

The study protocol is conformed to the ethical guidelines of the Declaration of Helsinki, and was reviewed and approved by the Ethical Committee of Kunming General Hospital of Chengdu Military Region. Statement of informed consent was obtained from all participants after full explanation of the procedure.

### Genotyping Assay

Genotyping experiments were performed as previously described [10]. Briefly, the blood samples were collected into tubes containing ethylenediaminetetraacetic acid and stored at −80°C until analysis. Standard phenol–chloroform extraction method was used to extract genomic DNA from whole blood. DNA concentration was measured by spectrometry (DU530 UV/VIS spectrophotometer, Beckman Instruments, Fullerton, CA, USA). Five SNPs in *HNMT*, *HDC* and *HRH3* (i.e., rs11558538, rs7164386, rs7182203, rs3787429 and rs3787430) were genotyped in this study by Sequenom MassARRAY RS1000 according to the standard protocol recommended by the manufacturer [13]. Sequenom MassARRAY Assay Design 3.0 Software was used to design Multiplexed SNP MassEXTEND assay [13], [14]. The primers used for the 5 SNPs are listed in Table S1. Sequenom Typer 4.0 Software was used to perform data management and analyses [13]–[15].

### Statistical Analysis

Statistical analyses were performed with SPSS 18.0 for Windows (PASW Statistics, SPSS Inc., Chicago, IL). A two-sided *P* value <0.05 was considered statistical significance for all tests. Bonferroni’s corrections were used for multiple comparisons. Each SNP frequency in control subjects was tested for departure from Hardy–Weinberg Equilibrium (HWE). Student *t* test, Chi-square (Pearson’s *χ* ^2^) test or Fisher’s exact test, and unconditional multivariate logistic regression analysis adjusted for age, menopausal state and body mass index (BMI) were used where necessary. Odds ratios (ORs) with 95% confidential intervals (CIs) were used to assess the associations between genotypes and breast carcinoma risk or clinical variables.

## Results

### Participants’ Characteristics

Descriptive statistics of both the case and control groups are given in Table 1, which shows significant difference regarding the BMI (*P* = 0.017) but no significant differences in age and the distribution of menopausal state (*P* = 0.262 and 0.611, respectively) between the 2 populations. Considering that variables of age, menopausal state and BMI may affect the development of breast cancer, these characters were adjusted further for any residual confounding effect in later multivariate logistic regression analyses. Most patients were diagnosed with clinical stage 1 or 2 (78.6%) and with ductal invasive carcinoma (87.6%) of breast origin. Furthermore, 58.0% of the patients were lymph node metastasis carriers.

**Table 1:** Characteristics of breast cancer patients and control participants.

	Breast Cancer (*n* = 201)	Control (*n* = 205)	*P*
Age (years)	46.5±9.2	45.6±7.0	0.262^a^
BMI (kg/m^2^)	23.1±2.9	22.4±2.6	**0.017** a
Sex			
Women	201 (100%)	205 (100%)	**–**
Menopausal state			
Premenopausal	126 (62.7%)	123 (60.0%)	0.611^b^
Postmenopausal	75 (37.3%)	82 (40.0%)	
Tumor size (cm)			
≤2.0	44 (21.9%)		
>2.0	157 (78.1%)		
Histology			
DIC	176 (87.6%)		
LIC	9 (4.4%)		
Others	16 (8.0%)		
Clinical stages			
I or II	158 (78.6%)		
III or IV	43 (21.4%)		
Lymph node metastasis			
Node-negative	116 (58.0%)		
Node-positive	85 (42.0%)		
Hormone receptor status			
Negative	56 (27.9%)		
Positive	145 (72.1%)		
HER2 ststus			
0–1	98 (48.8%)		
2–3	103 (51.2%)		

*BMI* body mass index, *DIC* ductal invasive carcinoma, *LIC* lobular invasive carcinoma.

a *P* values were calculated by student *t* tests.

b *P* values was calculated from two-sided chi-square test.

### Distributions of Genotype and Allele between Breast Cancer Patients and Health Controls

The distributions of genotype and allele frequencies of the 5 selected SNPs in breast cancer patients and health controls are listed in Table 2 and 3, respectively. We first performed HWE test, which revealed that none of the genotype distributions for the health controls differed significantly from those expected under HWE (*P* = 0.561 for rs11558538, 0.082 for rs7164386, 0.569 for rs7182203, 0.891 for rs3787429 and 0.056 for rs3787430). The minor allele frequencies (MAF) of the selected SNPs in control group were 0.039 for rs11558538, 0.095 for rs7164386, 0.090 for rs7182203, 0.324 for rs3787429 and 0.149 for rs3787430 (Table 3). In the following association analysis, we found that there were no significant associations between breast cancer risk and polymorphisms in *HNMT* and *HRH3* genotypes before or after adjustment for age, menopausal state and BMI (Table 2) in the present population, which were in accordance with the allele data (Table 3). As for *HDC* gene, we found that the frequency of the heterozygous variant CT genotype of rs7164386 in breast cancer case group was significantly differed from control group at Bonferroni-corrected *P* level of 0.01 (0.05/5 SNPs) before adjustment for age, menopausal state and BMI (unadjusted OR, 0.381; 95% CI, 0.193–0.753; *P* = 0.006) and that the difference still remain significant after further adjustment (adjusted OR, 0.402; 95% CI, 0.201–0.802; *P* = 0.009; Table 2). Furthermore, the T allele of rs7164386 also exhibited significantly different distributions between case and control groups and acted as protective effect against breast cancer (adjusted OR, 0.387; 95% CI, 0.208–0.720; *P* = 0.003; Table 3). While for rs7182203 in *HDC*, no association with breast cancer risk was observed according to both genotype and allele distributions data (Table 2 and 3).

**Table 2:** Frequency distributions of *HNMT*, *HDC* and *HRH3* genotypes and their associations with the risk of developing breast cancer.

Genotype	Breast Cancer	Control	Without Adjustment		With Adjustment	
	*n* (%)	*n* (%)	*P* a	OR (95% CI)	*P* b	OR (95% CI)
***HNMT***						
rs11558538						
CC	185 (92.0)	189 (92.2)		1.00 [Ref]		1.00 [Ref]
CT	16 (8.0)	16 (7.8)	1.000	1.022 (0.496–2.103)	0.970	0.986 (0.473–2.053)
TT	0 (0.0)	0 (0.0)	–	**–**	–	**–**
***HDC***						
rs7164386						
CC	187 (93.0)	170 (82.9)		1.00 [Ref]		1.00 [Ref]
CT	13 (6.5)	31 (5.1)	**0.006**	0.381 (0.193–0.753)	**0.009**	0.402 (0.201–0.802)
TT	1 (0.5)	4 (2.0)	0.199	0.227 (0.025–2.053)	0.195	0.229 (0.025–2.130)
rs7182203						
GG	170 (84.6)	169 (82.4)		1.00 [Ref]		1.00 [Ref]
GA	30 (14.9)	35 (17.1)	0.590	0.852 (0.500–1.451)	0.354	0.774 (0.450–1.331)
AA	1 (0.5)	1 (0.5)	1.000	0.994 (0.062–16.024)	0.728	0.609 (0.037–10.006)
***HRH3***						
rs3787429						
CC	79 (39.3)	94 (45.9)		1.00 [Ref]		1.00 [Ref]
CT	95 (47.3)	89 (43.4)	0.290	1.270 (0.838–1.925)	0.198	1.324 (0.863–2.031)
TT	27 (13.4)	22 (10.7)	0.260	1.460 (0.772–2.762)	0.270	1.440 (0.754–2.749)
rs3787430						
CC	143 (71.1)	152 (74.1)		1.00 [Ref]		1.00 [Ref]
CT	48 (23.9)	45 (22.0)	0.635	1.134 (0.711–1.808)	0.453	1.199 (0.746–1.929)
TT	10 (5.0)	8 (3.9)	0.632	1.329 (0.510–3.461)	0.697	1.215 (0.457–3.226)

*OR* odd ratio, *CI* confidence interval, *Ref* reference category.

Bonferroni’s multiple adjustment was applied to the level of significance, which was set at *P*<0.01 (0.05/5 SNPs).

a *P* values were calculated from two-sided chi-square tests or Fisher’s exact tests for either genotype distribution.

b *P* values were calculated by unconditional logistic regression adjusted for age, menopausal state and body mass index.

**Table 3:** Frequency distributions of *HNMT*, *HDC* and *HRH3* alleles and their associations with the risk of developing breast cancer.

Allele	Breast Cancer	Control	Without Adjustment		With Adjustment	
	*n* (%)	*n* (%)	*P* a	OR (95% CI)	*P* b	OR (95% CI)
***HNMT***						
rs11558538						
C	386 (96.0)	394 (96.1)		1.00 [Ref]		1.00 [Ref]
T	16 (4.0)	16 (3.9)	1.000	1.021 (0.503–2.070)	0.970	0.986 (0.481–2.023)
***HDC***						
rs7164386						
C	387 (96.3)	371 (90.5)		1.00 [Ref]		1.00 [Ref]
T	15 (3.7)	39 (9.5)	**0.001**	0.369 (0.200–0.680)	**0.003**	0.387 (0.208–0.720)
rs7182203						
G	370 (92.0)	373 (91.0)		1.00 [Ref]		1.00 [Ref]
A	32 (8.0)	37 (9.0)	0.616	0.872 (0.532–1.430)	0.338	0.782 (0.473–1.293)
***HRH3***						
rs3787429						
C	253 (62.9)	277 (67.6)		1.00 [Ref]		1.00 [Ref]
T	149 (37.1)	133 (32.4)	0.185	1.227 (0.918–1.638)	0.162	1.234 (0.919–1.656)
rs3787430						
C	334 (83.1)	349 (85.1)		1.00 [Ref]		1.00 [Ref]
T	68 (16.9)	61 (14.9)	0.444	1.165 (0.799–1.698)	0.411	1.174 (0.801–1.720)

*OR* odd ratio, *CI* confidence interval, *Ref* reference category.

Bonferroni’s multiple adjustment was applied to the level of significance, which was set at *P*<0.01 (0.05/5 SNPs).

a *P* values were calculated from two-sided chi-square tests or Fisher’s exact tests for either allele frequency.

b *P* values were calculated by unconditional logistic regression adjusted for age, menopausal state and body mass index.

For polymorphisms in *HDC* and *HRH3* in the present study, two independent tag-SNPs were selected from each gene. In order to further investigate their relationships with breast cancer risk, haplotype association analyses were performed. As shown in Table 4, for *HDC* haplotypes, the CG haplotype was more frequent among patients with breast cancer and may act as a potential risk factor of breast cancer after the adjustment (adjusted OR, 1.828; 95% CI, 1.218–2.744; *P* = 0.004); while the TG haplotype was more frequent among health controls and may be protecting factors (adjusted OR, 0.351; 95% CI, 0.182–0.678; *P* = 0.002). However, all the haplotypes of *HRH3* were found not to be significantly associated with breast cancer risk at the corrected *P* level of 0.00625 (0.05/8 haplotypes).

**Table 4:** Associations between risk of breast cancer and haplotypes of two *HDC* variants (rs7164386 and rs7182203) and two *HRH3* variants (rs3787429 and rs3787430).

Haplotypes^a^	Breast Cancer	Control	Without Adjustment		With Adjustment	
	*n* (%)	*n* (%)	*P* b	OR (95% CI)	*P* c	OR (95% CI)
***HDC***						
C-G	357 (88.8)	336 (82.0)	0.007	1.747 (1.172–2.605)	**0.004**	1.828 (1.218–2.744)
C-A	30 (7.5)	35 (8.5)	0.607	0.864 (0.520–1.436)	0.312	0.766 (0.457–1.285)
T-G	13 (3.2)	37 (9.0)	**0.001**	0.337 (0.176–0.644)	**0.002**	0.351 (0.182–0.678)
T-A	2 (0.5)	2 (0.5)	1.000	1.020 (0.143–7.276)	0.921	1.105 (0.153–7.981)
***HRH3***						
C-C	219 (54.5)	243 (59.2)	0.178	0.822 (0.623–1.086)	0.131	0.804 (0.606–1.067)
C-T	34 (8.5)	34 (8.3)	1.000	1.022 (0.622–1.679)	0.772	1.077 (0.651–1.783)
T-C	115 (28.6)	106 (25.9)	0.387	1.149 (0.843–1.566)	0.317	1.174 (0.857–1.609)
T-T	34 (8.4)	27 (6.6)	0.352	1.311 (0.775–2.216)	0.404	1.255 (0.736–2.137)

*OR* odd ratio, *CI* confidence interval.

Bonferroni’s multiple adjustment was applied to the level of significance, which was set at *P*<0.00625 (0.05/8 haplotypes).

a SNPs of haplotype are (in sequence) rs7164386 and rs7182203 for *HDC*, and rs3787429 and rs3787430 for *HRH3*, respectively.

b *P* values were calculated from two-sided chi-square tests or Fisher’s exact tests.

c *P* values were calculated by unconditional logistic regression adjusted for age, menopausal state and body mass index.

### Association between Genotype Distribution and Clinicopathological Parameters in Patients with Breast Cancer

Then, we further analyzed the genotype distributions of the 5 SNPs according to different clinicopathological parameters. Table 5 shows the correlations of clinicopathological parameters and the 2 *HDC* polymorphisms in breast cancer patients. There were no statistical significances on the associations between genotype distributions of rs7164386 or rs7182203 and all selected clinicopathological parameters at the corrected *P* level of 0.01 (0.05/5 SNPs) in the present study. Although the CT+TT genotypes of rs7164386 were a little more frequent in patients with low clinical stage (grades 1–2) or without lymph node metastasis, indicated by unadjusted *P* value (0.044 and 0.046, respectively) that were less than 0.05, there were eventually no association between the genotype distribution of rs7164386 and clinical stage after the adjustment (adjusted *P* = 0.249). However, the significance of the association between the genotype distribution of rs7164386 and lymph node metastasis still lay in a critical strip after the adjustment with *P* value (adjusted *P* = 0.037; OR, 0.193; 95% CI, 0.041–0.903) less than 0.05 but greater than 0.01 (the corrected *P* level) (Table 5). Additionally, the same analyses on the rest 3 SNPs (rs11558538, rs3787429 and rs3787430) were also performed with data shown in Table S2; and no statistically significant associations regarding these SNPs were observed in the present study.

**Table 5:** Correlations of clinicopathological parameters and *HDC* polymorphisms in patients with breast cancer.

	rs7164386			rs7182203		
	CC	CT+TT	*P* OR (95% CI)	GG	GA+AA	*P* OR (95% CI)
Age (years)	46.2±9.0	50.1±10.8	0.130^a^	46.4±9.2	47.0±9.1	0.754^a^
BMI (kg/m^2^)						
≥25	44 (93.6%)	3 (6.4%)	1.000^b^	37 (78.7%)	10 (21.3%)	0.248^b^
<25	143 (92.9%)	11 (7.1%)		133 (86.4%)	21 (13.6%)	
Menopausal state						
Premenopausal	120 (95.2%)	6 (4.8%)	0.151^b^	107 (84.9%)	19 (15.1%)	1.000^b^
Postmenopausal	67 (89.3%)	8 (10.7%)		63 (84.0%)	12 (16.0%)	
Tumor size (cm)						
≤2.0	39 (88.6%)	5 (11.4%)	0.194^b^	40 (90.9%)	4 (9.1%)	0.241^b^
>2.0	148 (94.3%)	9 (5.7%)		130 (82.8%)	27 (17.2%)	
Histology						
DIC	164 (93.2%)	12 (6.8%)	0.512^b^	148 (84.1%)	28 (15.9%)	1.000^b^
LIC	9 (100%)	0 (0.0%)		8 (88.9%)	1 (11.1%)	
Others	14 (87.5%)	2 (12.5%)		14 (87.5%)	2 (12.5%)	
Clinical stages						
Grade 1–2	144 (77.0%)	14 (23.0%)	0.044^b^	134 (84.8%)	24 (15.2%)	1.000^b^
Grade 3–4	43 (100%)	0 (0.0%)	0.249^c^; 0.294 (0.037–2.354)^d^	36 (83.7%)	7 (16.3%)	
Lymph node metastasis						
Node-negative	104 (89.7%)	12 (10.3%)	0.046^b^	99 (85.3%)	17 (14.7%)	0.844^b^
Node-positive	83 (97.6%)	2 (2.4%)	0.037^c^; 0.193 (0.041–0.903)^d^	71 (83.5%)	14 (16.5%)	
Hormone receptor status						
Negative	55 (98.2%)	1 (1.8%)	0.118^b^	49 (87.5%)	7 (12.5%)	0.523^b^
Positive	132 (91.0%)	13 (9.0%)		121 (83.4%)	24 (16.6%)	
HER2 ststus						
0–1	90 (91.8%)	8 (8.2%)	0.586^b^	84 (85.7%)	14 (14.3%)	0.700^b^
2–3	97 (94.2%)	6 (5.8%)		86 (83.5%)	17 (16.5%)	
p53 ststus						
Negative	49 (94.2%)	3 (5.8%)	0.828^b^	46 (88.5%)	6 (11.5%)	0.375^b^
Positive	75 (93.8%)	5 (6.3%)		69 (86.3%)	11 (13.7%)	
Undetermined	63 (91.3%)	6 (8.7%)		55 (79.7%)	14 (20.3%)	

*OR* odd ratio, *CI* confidence interval, *BMI* body mass index, *DIC* ductal invasive carcinoma, *LIC* lobular invasive carcinoma, *HER2* human epidermal growth factor receptor, *p53* tumor protein 53.

Bonferroni’s multiple adjustment was applied to the level of significance, which was set at *P*<0.01 (0.05/5 SNPs).

a *P* values were calculated by student *t* tests.

b *P* values were calculated from two-sided chi-square tests or Fisher’s exact tests.

c *P* values were calculated by unconditional logistic regression adjusted for age, menopausal state and body mass index.

d OR and 95% CI values were calculated by unconditional logistic regression adjusted for age, menopausal state and body mass index.

## Discussion

Gene polymorphisms of HDC, HNMT and HRH3 have already been found to result in expression and functional changes of the proteins and hence to be associated with many kinds of diseases [7]. Therefore, genes that encode HDC, HNMT and HRH3 are rationally considered as candidate genes associated with development of breast cancer that is believed to be a multiple gene-related disease and whose development was previously reported to be affected by these molecules [1], [3]–[5]. According to our present data, although no associations between polymorphisms of *HNMT* or *HRH3* and breast cancer were found, one polymorphism (rs7164386) of *HDC* did play certain roles to affect the disease in a Chinese Han population, which may be novel findings regarding the pathogenesis of breast cancer.

As for SNPs selection, rs7164386 and rs7182203 are tag-SNPs that captured the majority of known common variation in *HDC* while rs3787429 and rs3787430 are the only 2 tag-SNPs in *HRH3* according to data of Chinese Han population from HapMap (http://www.hapmap.org). Meanwhile, the rs11558538 in *HNMT* is also a generally investigated SNP in previous investigations [9], [16]–[18]. Therefore, these 5 SNPs were selected in our study. For characters of the control groups, the MAF of the selected SNPs in control group (Table 3) were close to the HapMap data (http://www.hapmap.org) and also previous studies on East Asian populations [8], [9]. Moreover, the HWE tests showed that the genotype distributions of these SNPs in control group were not deviate from HWE. Thus, the population in control group consists of a representative population sample for the present case-control study.

HDC is the key enzyme for histamine synthesis and alteration of its activity leads to changes of endogenous histamine levels. Previous investigations found that genetic polymorphisms of *HDC* contributed to certain kinds of histamine-related diseases such as allergic rhinitis and age at natural menopause [6], [19]. In line with these reports, our present study further observed the significant association between variants of rs7164386 in *HDC* and breast cancer risk according to both genotype and allele association analyses in current population, indicating that either this tag-SNP or other SNPs captured by rs7164386 within *HDC* alter the expression or function of HDC. More interestingly, the positive tag-SNP (rs7164386) found in the present study is located in the same linkage disequilibrium block as rs2073440 (See Figure S1), which is a nonsynonymous polymorphism and was found to be significantly associated with risk of developing rhinitis [19]. These findings strongly indicate that polymorphisms in this block of *HDC* may result in expression or functional changes of HDC although the underlying mechanisms need to be further elucidated. While for association analysis between rs7164386 and lymph node metastasis, the significance was not statistically meaningful after adjustment at the corrected *P* level. However, the OR (0.193) and 95% CI (0.041–0.903) values did indicate protecting effect of CT and TT variants against lymph node metastasis of breast cancer (Table 5). These findings are rational since endogenous histamine levels were found to be relevant to epithelial-to-mesenchymal transition and metastasis in cancer cells [20], [21]. Based on this background, the relationship between variants of *HDC* and metastasis of breast cancer may not be neglected. The relatively small sample size in our present study with increased type II error, which is an intrinsic limitation of the present study, may be one of the reasons for these results. Populations with larger sample sizes as well as meta-analyses are needed to further clarify this issue.

While for histamine degradation enzyme HNMT, we selected a nonsynonymous polymorphism rs11558538 in the present study. However, we did not find any significant associations between this SNP with breast cancer. One possible reason is that this nonsynonymous variant may not be strong enough to affect susceptivity of breast cancer in Chinese Han population although it is connected with lower HNMT activity both in vivo [22] and in vitro [23]. Another reason may be that HNMT is probably not the main histamine degradation enzyme in breast cancer tissues. Besides HNMT, diamine oxidase (DAO) is another enzyme involved in metabolism of histamine. It is reported that HNMT mainly catalyzes the inactivation of intracellular, while DAO scavenge extracellular histamine [9], [17]. Therefore, polymorphisms in *DAO*/*ABP1* gene may also contribute to breast cancer risk.

Previous studies found that the levels of HRH3 expression was significantly higher in breast cancer tissues compared with benign tissues and activation of HRH3 led to increased proliferation and migration of breast cancer cells [4], and that rs3787429 and rs3787430 were potential genetic markers for predicting the therapeutic effect of risperidone also in Chinese Han population [8]. It is noteworthy that the genotype distributions of these 2 tag-SNPs in the present study are in highly accordance with the previous report [8]. As *HRH3* is a relatively small gene (5.3 kb) and rs3787429 and rs3787430 are the only 2 tag-SNPs within *HRH3* according to HapMap data, it was our hope to find their associations with breast cancer. However, variants of rs3787429 and rs3787430 were found not to be associated with breast cancer, which indicates that polymorphisms of *HRH3* may either be minor variants for breast cancer or not affect the expression or function of HRH3. The molecular bases for these results are unclear yet and studies related to the effects of rs3787429 and rs3787430 on expression and activity of HRH3 are greatly warranted to clarify the underlying mechanisms.

In summary, our present study found for the first time that the variants of rs7164386 genotypes of *HDC* gene are significantly associated the risk of breast cancer while polymorphisms of *HNMT* and *HRH3* might be irrelevant with breast cancer in Chinese Han populations. The polymorphisms of *HDC* gene may be novel biomarker for personal treatment of breast cancer.

## Supporting Information

Figure S1 **Linkage disequilibrium plot of the SNPs in the HDC gene based on the measure of normalized linkage disequilibrium deviation according to data of Chinese Han population from HapMap.**
(TIF)

Table S1 Primers used for this study.
(DOC)

Table S2 Correlations of clinicopathological parameters and HNMT and HRH3 polymorphisms in patients with breast cancer.
(DOC)

## References

[1] Sieja K, Stanosz S, von Mach-Szczypiński J, Olewniczak S, Stanosz M (2005) Concentration of histamine in serum and tissues of the primary ductal breast cancers in women. Breast 14: 236–241. 15927833 10.1016/j.breast.2004.06.012

[2] Hegyesi H, Colombo L, Pállinger E, Tóth S, Boer K, et al (2007) Impact of systemic histamine deficiency on the crosstalk between mammary adenocarcinoma and T cells. J Pharmacol Sci 105: 66–73. 17895589 10.1254/jphs.fp0070636

[3] Medina V, Cricco G, Nuñez M, Martín G, Mohamad N, et al (2006) Histamine-mediated signaling processes in human malignant mammary cells. Cancer Biol Ther 5: 1462–1471. 17012845 10.4161/cbt.5.11.3273

[4] Medina V, Croci M, Crescenti E, Mohamad N, Sanchez-Jiménez F, et al (2008) The role of histamine in human mammary carcinogenesis: H3 and H4 receptors as potential therapeutic targets for breast cancer treatment. Cancer Biol Ther 7: 28–35. 17932461 10.4161/cbt.7.1.5123

[5] Santos RP, Benvenuti TT, Honda ST, Del Valle PR, Katayama ML, et al (2011) Influence of the interaction between nodal fibroblast and breast cancer cells on gene expression. Tumour Biol 32: 145–157. 20820980 10.1007/s13277-010-0108-7 PMC3003151

[6] Zhang F, Xiong DH, Wang W, Shen H, Xiao P, et al (2006) HDC gene polymorphisms are associated with age at natural menopause in Caucasian women. Biochem Biophys Res Commun 348: 1378–1382. 16919600 10.1016/j.bbrc.2006.08.008 PMC1803761

[7] García-Martín E, Ayuso P, Martínez C, Blanca M, Agúndez JA (2009) Histamine pharmacogenomics. Pharmacogenomics 10: 867–883. 19450133 10.2217/pgs.09.26

[8] Wei Z, Wang L, Zhang M, Xuan J, Wang Y, et al (2012) A pharmacogenetic study of risperidone on histamine H3 receptor gene (HRH3) in Chinese Han schizophrenia patients. J Psychopharmacol 26: 813–818. 21652606 10.1177/0269881111405358

[9] Lee HS, Kim SH, Kim KW, Baek JY, Park HS, et al (2012) Involvement of human histamine N-methyltransferase gene polymorphisms in susceptibility to atopic dermatitis in korean children. Allergy Asthma Immunol Res 4: 31–36. 22211168 10.4168/aair.2012.4.1.31 PMC3242058

[10] He GH, Lu J, Shi PP, Xia W, Yin SJ, et al (2013) Polymorphisms of human histamine receptor H4 gene are associated with breast cancer in Chinese Han population. Gene 519: 260–265. 23481304 10.1016/j.gene.2013.02.020

[11] González-Neira A (2012) Pharmacogenetics of chemotherapy efficacy in breast cancer. Pharmacogenomics 13: 677–690. 22515610 10.2217/pgs.12.44

[12] Justenhoven C, Obazee O, Brauch H (2012) The pharmacogenomics of sex hormone metabolism: breast cancer risk in menopausal hormone therapy. Pharmacogenomics 13: 659–675. 22515609 10.2217/pgs.11.144

[13] Gabriel S, Ziaugra L, Tabbaa D (2009) SNP genotyping using the Sequenom MassARRAY iPLEX platform. Curr Protoc Hum Genet 2: 12. 19170031 10.1002/0471142905.hg0212s60

[14] Li S, Jin T, Zhang J, Lou H, Yang B, et al (2012) Polymorphisms of TREH, IL4R and CCDC26 genes associated with risk of glioma. Cancer Epidemiol 36: 283–287. 22369735 10.1016/j.canep.2011.12.011

[15] Thomas RK, Baker AC, Debiasi RM, Winckler W, Laframboise T, et al (2007) High-throughput oncogene mutation profiling in human cancer. Nat Genet 39: 347–351. 17293865 10.1038/ng1975

[16] Marasović-Šušnjara I, Palada V, Marinović-Terzić I, Mimica N, Marin J, et al (2011) No association between histamine N-methyltransferase functional polymorphism Thr105Ile and Alzheimer’s disease. Neurosci Lett 489: 119–121. 21138759 10.1016/j.neulet.2010.11.078

[17] Maintz L, Yu CF, Rodríguez E, Baurecht H, Bieber T, et al (2011) Association of single nucleotide polymorphisms in the diamine oxidase gene with diamine oxidase serum activities. Allergy 66: 893–902. 21488903 10.1111/j.1398-9995.2011.02548.x

[18] Palada V, Terzić J, Mazzulli J, Bwala G, Hagenah J, et al (2012) Histamine N-methyltransferase Thr105Ile polymorphism is associated with Parkinson’s disease. Neurobiol Aging 33: 836.e1–3. 10.1016/j.neurobiolaging.2011.06.015 21794955

[19] Gervasini G, Agúndez JA, García-Menaya J, Martínez C, Cordobés C, et al (2010) Variability of the L-Histidine decarboxylase gene in allergic rhinitis. Allergy 65: 1576–1584. 20608921 10.1111/j.1398-9995.2010.02425.x

[20] Blaya B, Nicolau-Galmés F, Jangi SM, Ortega-Martínez I, Alonso-Tejerina E, et al (2010) Histamine and histamine receptor antagonists in cancer biology. Inflamm Allergy Drug Targets 9: 146–157. 20632959 10.2174/187152810792231869

[21] Meng F, Han Y, Staloch D, Francis T, Stokes A, et al (2011) The H4 histamine receptor agonist, clobenpropit, suppresses human cholangiocarcinoma progression by disruption of epithelial mesenchymal transition and tumor metastasis. Hepatology 54: 1718–1728. 21793031 10.1002/hep.24573

[22] Chen GL, Wang W, Xu ZH, Zhu B, Wang LS, et al (2003) Genotype-phenotype correlation for histamine N-methyltransferase in a Chinese Han population. Clin Chim Acta 334: 179–183. 12867290 10.1016/s0009-8981(03)00239-0

[23] Horton JR, Sawada K, Nishibori M, Zhang X, Cheng X (2001) Two polymorphic forms of human histamine methyltransferase: structural, thermal, and kinetic comparisons. Structure 9: 837–849. 11566133 10.1016/s0969-2126(01)00643-8 PMC4030376

