# Supplementary figures and images for: Associations of Polymorphisms in Histidine Decarboxylase, Histamine N-Methyltransferase and Histamine Receptor H3 Genes with Breast Cancer
Source: PLoS One. 2014 May 16;9(5):e97728. doi: 10.1371/journal.pone.0097728 (PMC4023951; doi:10.1371/journal.pone.0097728)

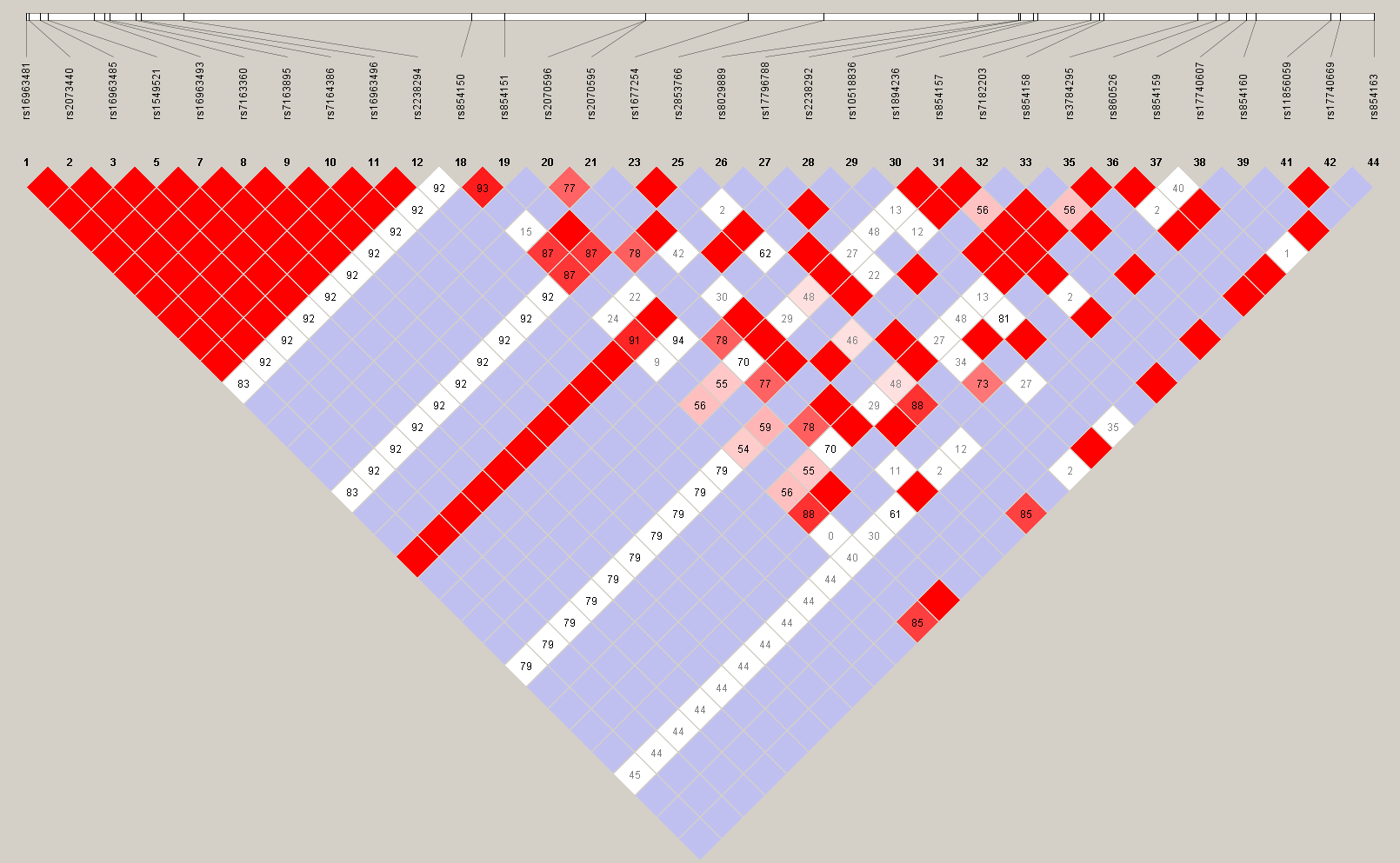

Supplement: Figure S1 — Linkage disequilibrium plot of the SNPs in the HDC gene based on the measure of normalized linkage disequilibrium deviation according to data of Chinese Han population from HapMap. (TIF) [file pone.0097728.s001.tif]
