# Supplementary material for: Associations of Polymorphisms in Histidine Decarboxylase, Histamine N-Methyltransferase and Histamine Receptor H3 Genes with Breast Cancer
Source: PLoS One. 2014 May 16;9(5):e97728. doi: 10.1371/journal.pone.0097728 (PMC4023951; doi:10.1371/journal.pone.0097728)
Supplement: Table S1 — Primers used for this study. (DOC) [file pone.0097728.s002.doc]

Table S1. Primers used for this study

| SNP_ID | Allele | 1st-PCRP | 2nd-PCRP | UEP_SEQ |
| --- | --- | --- | --- | --- |
| rs11558538 | C/T | ACGTTGGATGGCCAAGCAAACTTTACGTTC | ACGTTGGATGTGATGGTGTGTCACCTCTTC | GAGCTTGTAGCCAAGA |
| rs7164386 | C/T | ACGTTGGATGGATCTTCTGGATGCTTGGAG | ACGTTGGATGCCCAGGATTACTAACTAAAC | CCGGACAGGACTTGCCTT |
| rs7182203 | G/A | ACGTTGGATGATACCCCATAGGGACAGGTG | ACGTTGGATGCACGGGAAAAGTCAGGACAC | ACGTCAGGACACCACCCTGCT |
| rs3787429 | C/T | ACGTTGGATGAGAGGCCGCGCTCACTCAA | ACGTTGGATGGACACCATCTTCATGCGCTT | CATGCGCTTCTCCAG |
| rs3787430 | C/T | ACGTTGGATGGACACCATCTTCATGCGCTT | ACGTTGGATGAGAGGCCGCGCTCACTCAA | ATCAAGAGGAGCTCCAAGCC |
