# Supplementary material for: Associations of Polymorphisms in Histidine Decarboxylase, Histamine N-Methyltransferase and Histamine Receptor H3 Genes with Breast Cancer
Source: PLoS One. 2014 May 16;9(5):e97728. doi: 10.1371/journal.pone.0097728 (PMC4023951; doi:10.1371/journal.pone.0097728)
Supplement: Table S2 — Correlations of clinicopathological parameters and HNMT and HRH3 polymorphisms in patients with breast cancer. (DOC) [file pone.0097728.s003.doc]

Table S2. Correlations of clinicopathological parameters and *HNMT* and *HRH3* polymorphisms in patients with breast cancer

|  | rs11558538 | |  |  | rs3787429 | | |  | |  | | rs3787430 | | | |  | |  |
| --- | --- | --- | --- | --- | --- | --- | --- | --- | --- | --- | --- | --- | --- | --- | --- | --- | --- | --- |
|  | CC | CT + TT | *P*; OR (95% CI) |  | CC | CT + TT | | *P*; OR (95% CI) | |  | | CC | | CT + TT | | *P*; OR (95% CI) | |  |
| Age (years) | 46.5 ± 9.3 | 46.7 ± 7.6 | 0.929^a^ |  | 48.0 ± 9.5 | 45.5 ± 8.8 | | 0.058^a^ | |  | | 47.3 ± 9.1 | | 44.6 ± 9.1 | | 0.065^a^ | |  |
| BMI (kg/m^2^) |  |  |  |  |  |  | |  | |  | |  | |  | |  | |  |
| ≥25 | 40 (85.1%) | 7 (14.9%) | 0.062^b^ |  | 17 (36.2%) | 30 (63.8%) | | 0.733^b^ | |  | | 33 (70.2%) | | 14 (29.8%) | | 1.000^b^ | |  |
| <25 | 145 (94.2%) | 9 (5.8%) |  |  | 62 (40.3%) | 92 (59.7%) | |  | |  | | 110 (71.4%) | | 44 (28.6%) | |  | |  |
| Menopausal state |  |  |  |  |  |  | |  | |  | |  | |  | |  | |  |
| Premenopausal | 115 (91.3%) | 11 (8.7%) | 0.789^b^ |  | 49 (38.9%) | 77 (61.1%) | | 0.882^b^ | |  | | 88 (69.8%) | | 38 (30.2%) | | 0.632^b^ | |  |
| Postmenopausal | 70 (93.3%) | 5 (6.7%) |  |  | 30 (40.0%) | 45 (60.0%) | |  | |  | | 55 (73.3%) | | 20 (26.7%) | |  | |  |
| Tumor size (cm) |  |  |  |  |  |  | |  | |  | |  | |  | |  | |  |
| ≤2.0 | 43 (97.7%) | 1 (2.3%) | 0.203^b^ |  | 20 (45.5%) | 24 (54.5%) | | 0.384^b^ | |  | | 36 (81.8%) | | 8 (18.2%) | | 0.091^b^ | |  |
| >2.0 | 142 (90.4%) | 15 (9.6%) |  |  | 59 (37.6%) | 98 (62.4%) | |  | |  | | 107 (68.2%) | | 50 (31.8%) | |  | |  |
| Histology |  |  |  |  |  |  | |  | |  | |  | |  | |  | |  |
| DIC | 163 (92.6%) | 13 (7.4%) | 0.261^b^ |  | 69 (39.2%) | 107 (60.8%) | | 0.893^b^ | |  | | 124 (70.5%) | | 52 (29.5%) | | 0.219^b^ | |  |
| LIC | 7 (77.8%) | 2 (22.2%) |  |  | 3 (33.6%) | 6 (66.7%) | |  | |  | | 5 (55.6%) | | 4 (44.4%) | |  | |  |
| Others | 15 (93.8%) | 1 (6.3%) |  |  | 7 (43.8%) | 9 (56.3%) | |  | |  | | 14 (87.5%) | | 2 (12.5%) | |  | |  |
| Clinical stages |  |  |  |  |  |  | |  | |  | |  | |  | |  | |  |
| Grade 1-2 | 145 (91.8%) | 13 (8.2%) | 1.000^b^ |  | 61 (38.6%) | 97 (61.4%) | | 0.727^b^ | |  | | 109 (69.0%) | | 49 (31.0%) | | 0.255^b^ | |  |
| Grade 3-4 | 40 (93.0%) | 3 (7.0%) |  |  | 18 (41.9%) | 25 (58.1%) | |  | |  | | 34 (79.1%) | | 9 (20.9%) | |  | |  |
| Lymph node metastasis | |  |  |  |  |  | |  | |  | |  | |  | |  | |  |
| Node-negative | 109 (94.0%) | 7 (6.0%) | 0.294^b^ |  | 40 (34.5%) | 76 (65.5%) | | 0.110^b^ | |  | | 81 (69.8%) | | 35 (30.2%) | | 0.641^b^ | |  |
| Node-positive | 76 (89.4%) | 9 (10.6%) |  |  | 39 (45.9%) | 46 (54.1%) | |  | |  | | 62 (72.9%) | | 23 (27.1%) | |  | |  |
| Hormone receptor status | |  |  |  |  | |  | |  | |  | |  | |  | |  | |
| Negative | 52 (92.9%) | 4 (7.1%) | 1.000^b^ |  | 21 (37.5%) | | 35 (62.5%) | | 0.751^b^ | |  | | 41 (73.2%) | | 15 (26.8%) | | 0.731^b^ | |
| Positive | 133 (91.7%) | 12 (8.3%) |  |  | 58 (40.0%) | | 87 (60.0%) | |  | |  | | 102 (70.3%) | | 43 (29.7%) | |  | |

Table S2. continued

|  | | rs11558538 | |  |  | rs3787429 | |  |  | rs3787430 | |  |
| --- | --- | --- | --- | --- | --- | --- | --- | --- | --- | --- | --- | --- |
|  | | CC | CT + TT | *P*; OR (95% CI) |  | CC | CT + TT | *P*; OR (95% CI) |  | CC | CT + TT | *P*; OR (95% CI) |
| HER2 ststus |  | |  |  |  |  |  |  |  |  |  |  |
| 0-1 | | 87 (88.8%) | 11 (11.2%) | 0.120^b^ |  | 39 (39.8%) | 59 (60.2%) | 1.000^b^ |  | 66 (67.3%) | 32 (32.7%) | 0.277^b^ |
| 2-3 | | 98 (95.1%) | 5 (4.9%) |  |  | 40 (38.8%) | 63 (61.2%) |  |  | 77 (74.8%) | 26 (25.2%) |  |
| p53 ststus | |  |  |  |  |  |  |  |  |  |  |  |
| Negative | | 48 (92.3%) | 4 (7.7%) | 0.345^b^ |  | 19 (36.5%) | 33 (63.5%) | 0.109^b^ |  | 35 (67.3%) | 17 (32.7%) | 0.452^b^ |
| Positive | | 76 (95.0%) | 4 (5.0%) |  |  | 26 (32.5%) | 54 (67.5%) |  |  | 55 (68.8%) | 25 (31.2%) |  |
| Undetermined | | 61 (88.4%) | 8 (11.6%) |  |  | 34 (49.3%) | 35 (50.7%) |  |  | 53 (76.8%) | 16 (23.2%) |  |

*OR* odd ratio, *CI* confidence interval, *BMI* body mass index, *DIC* ductal invasive carcinoma, *LIC* lobular invasive carcinoma, *HER2* human epidermal growth factor receptor, *p53* tumor protein 53

Bonferroni’s multiple adjustment was applied to the level of significance, which was set at *P* < 0.01 (0.05/5 SNPs)

^a^ *P* values were calculated by student *t* tests

^b^ *P* values were calculated from two-sided chi-square tests or Fisher’s exact tests

^c^ *P* values were calculated by unconditional logistic regression adjusted for age, menopausal state and body mass index

^d^ OR and 95 % CI values were calculated by unconditional logistic regression adjusted for age, menopausal state and body mass index
